# Monthly Memoranda

**Published:** 1853-12

**Authors:** 


### EDITORIAL DEPARTMENT.

---

MONTHLY MEMORANDA.—*Ice, employed as a Local Anæsthetic*, has of late attracted considerable attention. The French Surgeons have used a mixture of powdered ice and salt, for the purpose of destroying the sensibility of a part upon which an operation was about to be performed. The ice is finely powdered and mixed with dry salt, enclosed in a bag of oiled silk, and applied to the part. A few minutes of course suffice to render the part insensible to pain. From the very nature of the case, such an application would be suitable only to quite limited portions of the body, and to operations upon the fingers and toes: it would be peculiarly applicable in those exceedingly painful operations, the removal of the nails. Another advantage, in addition to the relief from pain, by such an application, might be the prevention of excessive hemorrhage.

*Sick Headache.*—Dr. Folsom, of Portsmouth, N. H., recommends the following treatment, in this distressing complaint:

"Take any number of drops of croton oil, mix them with flour and molasses, and make as many pills as drops of the oil used. When the patient begins to feel the sick headache coming on, one half of a pill is to be taken every hour in molasses, or something of like consistence, until it acts as a cathartic; and thus treat the sick headache at each attack. If thus taken, each attack will be less severe, and in some cases a few doses produce a permanent cure. He seems to think the croton oil acts in three ways: 1st. By increasing the secretions. 2d. By counteracting the anti-peristaltic action of the stomach and bowels; and 3d, by acting as a counter-irritant to the brain.

*Habitual Constipation* is the subject, as the reader will have noticed, of one of our selected articles. We must beg leave to differ somewhat from the learned author of the article, and to still think, as we always have done, that few evils are so common, and so prejudicial to the general health as that alluded to. The functions of the alimentary organs are too important, for a state of absolute health to exist, during their entire neglect. The few cases cited, where an almost entire suspension of intestinal action seemed compatible with general health, are merely physiological curiosities: so are opium-eating, arseniceating, and habitual intoxication and—Barnum's wooly horse. We fear, too, that we should differ again with Christison in regard to the continuous treatment of habitual constipation. We should demur from the long continued use of laxatives, year after year, fearing lest such *artificial* means, once fairly commenced, can never be suspended. We should rather insist upon some *natural* substitute, such as the partial use of coarse food, corn bread, wheaten grits, hominy, and of ripe fruits, including the coarse skin and rinds. Sedentary habits are a frequent cause of this evil, and to many persons a walk of two or three miles is as good as a dose of rheubarb. Many other methods will suggest themselves to the physician, such as kneading the abdomen, voluntary solicitation and aqueous enemata. More attention should be paid to this subject, especially in those portions of the country where the character of the prevailing diseases is so generally "bilious"—a word we wish we could avoid.

*Cholera Infantum* is said by the editors of the Virginia Med. and Surg. Journal, to be very successfully treated by the use of sub-nitrate of bismuth. During the past summer they have had occasion to employ this remedy in a considerable number of cases, and have had every reason to be satisfied with its effects. It is best administered to children in suspension, as by the following formula: Subnitrate of bismuth, two drachms; gum acacia, one drachm; orange flower water and simple syrup, each one ounce; water, two ounces: a teaspoonful every hour. They have found the anti-vomitive power of this remedy to be very remarkable.

*A case of poisoning*, by laudanum, is reported by Dr. Schaffer, in the American Lancet. Miss S., aged 23, had taken an ounce and a half of laudanum. When seen by Dr. S. she was in profound coma, and had been so for twenty minutes; pulse scarcely perceptible; respiration slow and stertorous; mouth and nostrils livid. An emetic of sulph. zinc was prepared, but the jaws were found firmly set. What was to be done? A tub of cold water was brought, her head held over it, and a large stream poured coutinuously over her forehead and face. She soon aroused with a tremendous struggle, and the emetic was administered. She soon relapsed, but the water roused her again. The emetic took effect—the cold water was repeated, for some hours, during her sleepy intervals, and her convalescence was favorable. Another interesting case will be found among our selections, in which the usual emetic failed of stimulating the stomach, when a somewhat novel expedient of administering alum was resorted to. It would be difficult to state how much the vomiting was due to alum, and how much to the distension of the stomach by copious draughts of warm water.

*New Method in Fractures.*—Dr. Williams, of Cincinnati, writes from Paris, to the Western Journal, of a method of applying an immovable apparatus, for fractures, lately introduced into practice there, of which he speaks favorably. The many-tailed bandage of Scultetus is prepared and wet with water. A portion of plaster of Paris, such as is commonly used in stucco work, thoroughly dried for the occasion, is sprinkled upon each strip, which is instantly applied. In a few seconds the whole bandage is perfectly dry and solid—as soon as the stucco *sets.* The advantages are that, drying instantly, it maintains its own extension and counter-extension, and the limb cannot easily shorten. To prevent irritation of the skin, the limb should be enveloped in a roller before the application of the plaster.

*Lethean Liniment* is the very appropriate name, given by Dr. Douglass, to a new combination, published by him in the Southern Medical and Surgical Journal: "It is made by digesting a bar of fresh turpentine soap and four ounces of gum camphor in a gallon of good alcohol for two weeks in the heat of the sun. It is then bottled up while hot, and one drachm of chloroform added to every four ounces, set in a cool place, and shaken occasionally while coagulating.

"The turpentine affords," says Dr. D., "the best means, in my opinion, of applying chloroform to the skin, because, by its adhesiveness it holds that volatile fluid longer and more firmly in contact with the surface than any other substance could do.

"My mode of applying it, is to coat the part well with the liniment and cover it immediately with a piece of good paper, which adheres firmly and produces a gentle burning, tingling sensation, which, in neuralgia, rheumatism, irritability of the stomach, cramp colic, &c., is perfectly delightful."

*Vaccination in Hooping Cough.*—A case of some interest occurred in our practice, some months since, which seems to merit notice. A fine healthy boy, aged about two years, was seized with hooping cough, which soon assumed a violent and severe character. In spite of the most prompt and judicious treatment, it rapidly became worse, until the paroxysms of coughing put on that fearful and alarming character so well known to those familiar with the severest forms of this disease. The most decided means were necessary to interrupt the continuance of the paroxysm. Dashing cold water was of necessity employed; the gasping for breath and apparently impending asphyxia gave to each paroxysm the appearance of the last. The case had gone on, from bad to worse, for five weeks, when the extreme prostration of the little sufferer caused and aggravated by his paroxysms, gave little hope for the future. At this time, the sthenic stage of the disease having subsided, it was thought a favorable opportunity to employ vaccination. It was done, and from the very day that the constitutional effect of the vaccination was produced, the cough abated—the violence of the nervous action immediately subsided, and the case terminated at once in a most welcome recovery.

